# The Effectiveness of Digital Therapeutics Intervention in Oral Anticoagulation Management: A Systematic Review and Meta-analysis

**DOI:** 10.1016/j.mcpdig.2026.100336

**Published:** 2026-01-20

**Authors:** Jiayue Guo, Lili You, Lu Liu, Xitong Jiao, Debasish Kar, Jitendra Jonnagaddala

**Affiliations:** a School of Health Policy and Management, Peking Union Medical College, Beijing, China; b Community and Primary Care Research Group, University of Plymouth, United Kingdom; c Nuffield Department of Primary Care Health Sciences, University of Oxford, United Kingdom; d Discipline of General Practice, School of Clinical Medicine, UNSW Sydney, Kensington, Australia; e SREDH Consortium, Sydney, Australia

## Abstract

**Objective:**

To summarize the key intervention characteristics and evaluate the effectiveness and safety of digital therapeutics (DTx) in patients receiving oral anticoagulation, with effectiveness evaluated using time in therapeutic range (TTR), thromboembolic events, and mortality, and safety evaluated based on bleeding events.

**Patients and Methods:**

We searched PubMed, Embase, Web of Science, and the Cochrane Library from inception to June 20, 2025, and identified 10 randomized controlled trials involving 7237 patients. The criteria required studies to assess software-based DTx supporting anticoagulation management and report effectiveness or safety outcomes. Study quality was evaluated using the Grading of Recommendations, Assessment, Development, and Evaluation framework, and random-effects models were applied.

**Results:**

Digital therapeutics interventions were associated with a lower incidence of major bleeding than usual care: no clear differences in TTR, thromboembolic events, or mortality. Evidence quality ranged from very low to high. Secondary analyses showed more international normalized ratio testing with DTx; rehospitalization rates did not differ significantly between the groups. Sensitivity analysis changed TTR effect after excluding a study with enhanced control, but other outcomes remained unchanged.

**Conclusion:**

Digital therapeutics interventions for anticoagulation management improve safety outcomes, particularly reducing major bleeding, and with greater monitoring intensity. Larger, long-term trials are needed to confirm the clinical benefits and evaluate cost-effectiveness.

**Trial Registration:**

PROSPERO Identifier: CRD420251107441.

Oral anticoagulation (OAC) therapy is the cornerstone of treatment for several diseases and has been prescribed to millions of people worldwide. Atrial fibrillation (AF), pulmonary embolism, deep vein thrombosis (DVT), and postoperative heart valve replacement are the most common indications. To achieve the therapeutic goal, adherence to therapy and its monitoring are crucial.^1^^,^^2^ Over the last decades, anticoagulation therapy has evolved considerably, expanding from the long-standing use of warfarin to include direct oral anticoagulants (DOACs). Old age, poor renal function, multimorbidity, polypharmacy, and drug interaction are major considerations in prescribing DOAC,^3^ leading to an increasing complexity of medical therapy.^4^ Warfarin has the advantage of being inexpensive and having a wide range of indications but requires careful monitoring and dose titration owing to a narrow therapeutic window, drug and food interactions, and bleeding complications.^5^ Clinically, warfarin dosage adjustment requires careful international normalized ratio (INR) monitoring because it indicates the therapeutic effect on reducing thromboembolic and bleeding risks.^6^

Advances in testing and communication technologies have facilitated the use of self-testing and self-management in routine clinical practice.^7^^,^^8^ Portable coagulometers allow patients to measure their INR within minutes using fingertip blood samples without the need to visit hospital clinics,^9^ supporting a more community-based model of care. Several review articles have evaluated the effectiveness of digital health interventions, including telemedicine platforms, remote monitoring systems, telephone or voicemail follow-up, SMS messaging, mobile applications, and other telehealth tools for promoting patient satisfaction, medication adherence, anticoagulation control, and economic viability.^10^, ^11^, ^12^, ^13^, ^14^, ^15^, ^16^ However, most of these interventions primarily support or extend existing care delivery by adjusting communication, monitoring, or care coordination. They do not, by themselves, deliver a continuous, patient-level therapeutic effect. Moreover, many mobile health interventions are developed with limited clinician involvement, potentially constraining their integration into routine clinical practice.

As defined by the Digital Therapeutics Alliance, digital therapeutics (DTx) are software-driven interventions with an explicit therapeutic intent and claim, in which the software itself delivers an active intervention to treat, manage, or prevent disease.^17^ Consistent with Software as a Medical Device principles, such interventions should be supported by a structured analytical and clinical validation and appropriate lifecycle controls,^18^^,^^19^ whereas health-system frameworks^20^ align evidence expectations with claimed function and risk. Previous studies have assessed DTx effectiveness in cognitive disorders, hypertension management, and other diseases.^21^, ^22^, ^23^, ^24^ Unlike broader digital health tools, qualifying DTx are characterized by a closed-loop therapeutic mechanism in which patient data are algorithmically processed to generate individualized therapeutic actions.^25^ Most existing digital anticoagulation technologies would not meet DTx standards. They are clinician-facing digital education and training modules aiming to improve knowledge and confidence^26^; remote INR solutions extending measurement and reporting^27^; SMS reminders providing prompts to support adherence^28^; computer-assisted dosing algorithms and clinical decision support systems (CDSSs) improving dosing consistency or time in therapeutic range (TTR)^29^, ^30^, ^31^, ^32^; telehealth and data-transmission systems functioning as service-delivery infrastructure.^14^^,^^33^^,^^34^

Additionally, several patient-facing tools provide software-based education, adherence–support, or decision support, such as WhatsApp-based INR management,^35^ the Health Buddies application,^36^ ACAFiB-APP,^37^ and other software systems,^38^, ^39^, ^40^ but most have so far lacked randomized controlled trial (RCT) validation and have yet to be formally recognized or reimbursed as DTx products for anticoagulation management.

Owing to the ambiguity of the necessary attributes required for DTx products and the lack of high quality trials, evidence on the DTx approach has not yet been synthesized, and the pooled quantitative effect remains unclear. Therefore, this meta-analysis aimed to identify and synthesize evidence from RCT comparing the effects of DTx with those of usual care in patients receiving anticoagulant therapy.

## Patients And Methods

This study was performed in accordance with the Cochrane Collaboration guidelines^41^ and PRISMA statement^42^ (Supplemental Table 1, available online at https://www.mcpdigitalhealth.org/). The GRADE (Grading of Recommendations, Assessment, Development, and Evaluation) was used to assess the quality of the evidence.^43^ The protocol was registered in PROSPERO, CRD420251107441.^44^ Preliminary results were presented at the ISPOR Advancing Patient-centered Research^45^ and have also been made available as a preprint.^46^ Given that our study focused on systematic review and meta-analysis, there was no need for ethical review board approval or obtaining informed consent from the participants.

### Search Strategy and Selection Criteria

Four databases (PubMed, Embase, Web of Science, and the Cochrane Library) were searched from their inception through June 20, 2025, with no language or publication type restrictions. For the PubMed search, we used the following search terms: (*telemedicine* OR *mhealth* OR *mobile health* OR *digital health* OR *smartphone application* OR *eHealth* OR *telecommunication* OR *telemonitoring* OR *digital therapeutics*) AND (*anticoagulants* OR *heparin* OR *warfarin* OR *DOAC* OR *anticoagulant agent* OR *anticoagulation* OR *anticoagulation management*) OR (*venous thromboembolism* OR *deep vein thrombosis* OR *pulmonary embolism* OR *atrial fibrillation*). The search terms were chosen to be sufficiently inclusive to identify any study in which DTx served as an intervention for anticoagulation management, in whole or in part. The complete list of search terms is provided in Supplemental Appendix (available online at https://www.mcpdigitalhealth.org/). Reference lists from related original articles and reviews were also investigated. To ensure a comprehensive search, we scanned a clinical research database (http://clinicaltrials.gov/) and the product library of the Digital Alliance (http://dtxalliance.org/understanding-dtx/product-library/) to obtain current clinical trials and products related to OAC DTx. Two reviewers (J.G. and L.Y.) independently screened all titles and abstracts to determine eligibility for inclusion and read the full-text of the remaining articles. In cases of disagreement, a third author (J.J.) was consulted to reach consensus.

We included all software-based interventions that met our operational DTx criteria, irrespective of their formal regulatory approval status. The following inclusion criteria were applied: (1) RCTs; (2) patients on warfarin therapy or DOACs for a single disease or multiple conditions; (3) digital intervention meeting the following DTx criteria, which can be standalone or combined with self-help therapies or hardware-assisted therapies; (4) comparison group receiving conventional anticoagulation management, usual care, or standard follow-up; and (5) reported outcomes on effectiveness and safety. Effectiveness outcomes included TTR, thromboembolism events (TEEs), and mortality, whereas safety outcomes included bleeding events.

Authorization or market approval status was not used as an inclusion criterion because eligible products and trials in anticoagulation management are currently limited, and restricting eligibility to authorized products may preferentially include interventions that have already undergone regulatory review, thereby limiting generalizability. Therefore, we focused on interventions that demonstrated mechanism features consistent with the conceptual definition of digital therapeutics.

According to the definition and core principles of the Digital Therapeutics Alliance, the criteria for DTx intervention were explicated in this study as follows: (1) software-driven therapeutic module: the intervention need to be implemented as a software, such as smartphone applications, web-based platform, or integrated clinical software, and intended to directly influence anticoagulation management, either as a standalone or in concert with other treatments; (2) structured patient-level data input: the software requires users (patient and/or clinicians) to enter individual clinical or treatment data relevant to anticoagulation, such as current oral anticoagulant regimen, INR or other laboratory values, comorbidities, symptoms, or risk factors; (3) embedded clinical logic: the software incorporated an explicit decision logic, such as dosing algorithms, risk stratification rules, or predictive models, which have been specified by or developed in collaboration with health professionals and which process the input data to generate clinical meaningful recommendations; and (4) personalized therapeutic output, such as tailored dosing advice, safety alerts, follow-up schedules, behavior-change tasks, or educational messages, to support anticoagulation therapy or disease management; and (5) clinical involvement and workflow integration: the software provides clinician access for reviewing patient data, validating or adjusting recommendations, and coordinating follow-up, ensuring routine clinical care integration.

The digital tools ineligible for DTx intervention include the following: (1) nontherapeutic tools that facilitate communication, monitoring, data transfer, or reminders; (2) non–patient-facing systems providing static information, generic education, or uniform reminders; (3) systems lacking embedded clinical logic or clinical outcome validation, such as those without explicit dosing algorithms, rule sets, risk models, or evaluated only for feasibility/acceptability without clinical outcomes or randomized trial evidence; and (4) clinician-only utilities without patient-level therapeutic delivery, such as decision support or administrative tools that provide guideline text or alerts but do not generate individualized therapeutic outputs.

The exclusion criteria were as follows: (1) digital-based interventions that did not meet the criteria for DTx; (2) nonrandomized and observational studies or unavailable full-text publications; (3) studies without a control group; (4) repeated reports; and (5) review articles, editorials, guides, case reports, or conference literature without RCT findings.

### Statistical Analyses

Data extraction was performed by J.G. and checked by J.J. using a standardized data extraction form implemented in Microsoft Excel. Data included information on the study author (year), population, intervention and control components, follow-up time, type of anticoagulant, type of disease, and outcomes. When the required study information or data were missing from the publication, the corresponding authors were contacted via email. Risks of bias were assessed by J.G. and reviewed by J.J. using the Cochrane Collaboration risk of bias tool.^47^

The primary outcomes were TTR, major bleeding, TEE, and mortality. The secondary outcomes included the frequency of testing, rehospitalization, management costs, and medication adherence. A meta-analysis of RCTs was conducted in RevMan (version 5.4; Cochrane) using random-effects models. Mean differences (MDs) were calculated for continuous outcomes and pooled relative risks for binary outcomes, with their respective 95% CIs. We used a random-effects model because of the clinical heterogeneity of the included studies (types of DTx interventions and population characteristics). Statistical heterogeneity was quantified using the χ^2^ and *I*^2^ statistical tests. Statistical heterogeneity was considered important when *I*^2^ was greater than 50%, the *P* value of the χ^2^ test was less than .05, and studies differed in both the magnitude and direction of the effects. For multiarm trials, the shared control group was allocated equally across comparisons to avoid double-counting participants. Publication bias was assessed using funnel plots and Egger test. However, the small number of studies (n=10) included in the meta-analysis precluded such investigations. Sensitivity analysis was conducted by excluding each study at a time and reanalyzing the remaining data sets to assess the stability and reliability of the results. Subgroup analyses were performed across DTx delivery modalities and study populations to explore between-study heterogeneity.

## Results

### Study and Participant Characteristics

**Figure 1:**
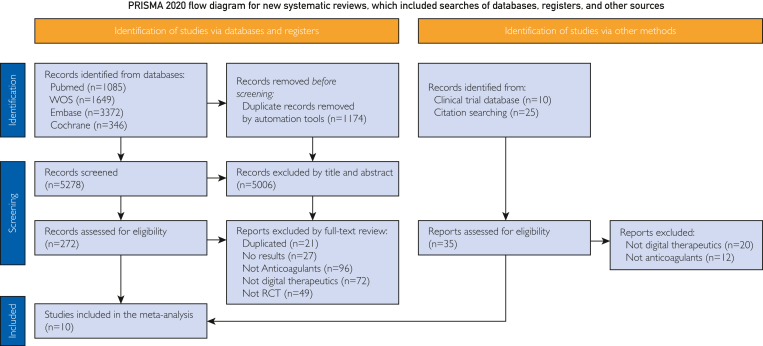
PRISMA flow diagram of study selection. RCT, randomized controlled trial.

The database search yielded 6452 articles and 35 additional references from the clinical trial database and reference list. After removing duplicates, we screened the titles and abstracts of 5278 references and retained 272 references for a full-text review. Ten RCTs were included in this meta-analysis (Figure 1).

Table 1^47^, ^48^, ^49^, ^50^, ^51^, ^52^, ^53^, ^54^, ^55^, ^56^, ^57^ summarizes the main characteristics of the 10 included studies conducted between 2009 and 2025. Seven studies were conducted since 2020 and 3 between 2009 and 2019. Five (50%) studies were conducted in China^48^, ^49^, ^50^, ^51^, ^52^ and 1 (10%) in Ireland,^53^ Germany,^54^ Canada,^55^ Denmark,^56^ and Korea,^57^ involving 7237 patients. They included 1 (10%) cluster RCT,^55^ 1 (10%) crossover trial,^53^ and 8 (80%) parallel-group RCTs, with 3794 patients in the DTx group and 3443 in usual care. One study had 2 independent intervention comparisons based on INR ranges, labeled Koertke a and Koertke b.^54^ Sample sizes ranged from 87 to 1890, and study duration varied from 3 to 12 months. Among the enrolled patients, 3413 (47.2%) underwent cardiac valve replacement and received long-term warfarin therapy, 3617 (50.0%) had AF and received vitamin K antagonist or DOAC therapy, and 207 (2.9%) patients had mixed indications for warfarin therapy. All trials included diverse groups of patients with hypertension, diabetes mellitus, AF, vascular diseases, or thromboembolic risks. The mean age of study participants ranged from 49.6 to 72.5 years. None of the interventions reported obtaining regulatory approval or reimbursement as prescription DTx.

**Table 1:** Characteristics of included RCTs

Country	DTx modality	DTx type	Sample size, n	Follow up months	Population	Other diseases	Anticoagulant type	Average age (SD)	Men, n (%)	TTR, mean % (SD)	Major bleeding, n (%)	Thromboembolism events, n (%)	All cause death, n (%)	Other outcomes
Ireland^53^	Internet-based expert system plus home INR self-testing	Warfarin dose-adjustment / INR-guided self-management systems	60 avs 60	12.0	Patients on long-term warfarin therapy	AF, prosthetic heart valve, DVT/PE	Warfarin	58.7 (14.3)	80 (61.6%) overall	74.0 (12.15) vs 58.6 (23.7)	0 (0.0) vs 1 (1.7)	2 (3.3) vs 1 (1.7); TIA, DVT	..	Frequency of INR testing: 4.6-day vs 19.6-day intervals
Germany^54^	Internet-based telemedicine-guided INR self-control system	Warfarin dose-adjustment / INR-guided self-management systems	521 vs 524 vs 526	6.0	Patients after MHVR (aortic/mitral/double valves) for valvular heart disease	Diabetes mellitus, hypertension, previous MI, peripheral arterial occlusive disease	Warfarin (VKA)	57.3 (11.9) vs 56.7 (11.9) vs 59.2 (10.9)	374 (71.8%) vs 393 (74.9%) vs 356 (67.7%)	77.5 (15.0) vs 77.6 (13.7) vs 83.9 (12.7)	5 (1.4%) vs 5 (0.9%) vs 19 (3.7%)	≈1 (0.2%) vs 6 (1.1%) vs 5 (1.0%); ischaemia, stroke, TIA, stenosis, MI	7 (1.2%) vs 15 (2.9%) vs 6 (1.1%); sepsis, tumour, CD, aortic aneurysm, CF, HF, accident, MOF	Long-term composite events and INR control
Denmark^56^	Telemedicine software linked to INR test device	Warfarin dose-adjustment / INR-guided self-management systems	44 vs 43	10.0	Patients on warfarin therapy eligible for self-testing/management	AF, DVT, valvular heart disease, cardiomyopathy, aneurysm, thrombophilia, stroke	Warfarin	68.9 vs 69.9	35 (79.5%) vs 34 (79.0%)	82.7 (12.0) vs 81.6 (15.8)	0 (0) vs 0 (0)	..	0 (0) vs 0 (0)	Frequency of INR testing: 7-day vs 7-day intervals; healthcare professional interactions
Canada^55^	Internet-based clinical decision support system for AF	Integrated AF management pathways with embedded anticoagulation components	590 vs 543	12.0	AF patients managed in primary care	HF, hypertension, stroke, TIA, systemic embolism, vascular disease	Warfarin; DOAC	72.1 (9.9) vs 72.5 (10.1)	350 (64.5%) vs 351 (59.5%)	..	8 (1.3%) vs 7 (1.3%); fatal bleeding 2 (0.3%) vs 0 (0.0%)	6 (6.3%) vs 3 (4.1%); TIA/stroke, SE, ischaemia, ACS	28 (4.7%) vs 21 (3.9%); HF, syncope/presyncope, stroke, ischaemic causes	Rehospitalization: 40 (6.7%) vs 34 (6.3%)
China^48^	Mobile app plus internet-based warfarin management	Warfarin dose-adjustment / INR-guided self-management systems	360 vs 361	12.0	Patients after MHVR for valvular heart disease	Hypertension, AF, angina	Warfarin	49.59 (9.46) vs 50.6 (9.65)	220 (60.9%) vs 220 (61.1%)	Mean TTR: 53 (24) vs 46 (21)	General bleeding: 22 (6.11%) vs 40 (11.08%); major bleeding: 2 (0.56%) vs 4 (1.11%); all bleeding: 24 (6.67%) vs 44 (12.19%)	All embolic events: 1 (0.28%) vs 2 (0.55%); neurologic embolic: 1 (0.28%) vs 0; noncerebral embolic: 0 vs 1 (0.28%)	0 (0) vs 2 (0.55%)	Rehospitalization: 4 (1.11%) vs 6 (1.66%)
China^49^	Mobile social app (WeChat mini-program)	Warfarin dose-adjustment / INR-guided self-management systems	362 vs 356	12.0	Patients after MHVR for valvular heart disease	AF, diabetes, hypertension, stroke, coronary artery disease, peripheral arterial disease, hepatic dysfunction, renal dysfunction	Warfarin	50.3 (9.8) vs 51.3 (9.5)	144 (39.8%) vs 144 (40.4%)	71.5 (14.6) vs 52.6 (12.9)	9 (1.25%) vs 21 (2.92%)	12 (1.67%) vs 15 (2.09%); valve thrombosis, MI, AT, VT, peripheral embolism, PE, stroke	0 (0) vs 0 (0)	Frequency of INR testing: 26.0 (8.57) vs 20.1 (6.47)
China^50^	Mobile app with researcher network implementing ABC pathway	Integrated AF management pathways with embedded anticoagulation components	833 vs 1057	12.0	AF patients with multimorbidity	Hypertension, coronary artery disease, HF, cardiomyopathy, peripheral arterial disease, diabetes, liver/kidney dysfunction, pulmonary disease, stroke	Warfarin; DOAC	72.0 (12.0) overall	555 (67.6%) vs 667 (58.1%)	..	0 (0) vs 9 (0.8%); all bleeding 22 (2.6%) vs 47 (4.4%)	4 (0.5%) vs 31 (2.9%); stroke, systemic embolism	12 (1.4%) vs 48 (4.5%)	Rehospitalization: 33 (4.0%) vs 116 (11.0%)
China^51^	Mobile app (Alfalfa)	Patient-facing education and adherence–support applications	48 vs 48	12.0	AF patients with stroke risk factors	Hypertension, diabetes, HF	VKAs; DOAC	61.65 (11.01); 61.52 (9.96) vs 61.77 (12.09)	20 (41.7%) vs 17 (35.4%)	..	OAC bleeding: 8 (16.7%) vs 10 (20.8%); DOAC bleeding: 7 (18.0%) vs 7 (17.5%)	1 (2.1%) vs 0 (0)	..	Anticoagulation knowledge: 78.1% vs 25.0%; medication adherence improvements
China^52^	Mobile app (Alfalfa) with Internet + pharmacy care model	Warfarin dose-adjustment / INR-guided self-management systems	204 vs 201	3.0	Patients after cardiac valve replacement (biological or mechanical)	Hypertension, diabetes, hyperuricemia or gout	Warfarin	49.66 (12.37) vs 51.97 (13.58)	115 (56.4%) vs 101 (50.2%)	66.46 (25.22) vs 46.65 (39.13); p<0.001	Minor bleeding: 12 (5.9%) vs 3 (1.5%); major bleeding: 3 (1.5%) vs 1 (0.5%)	1 (0.5%) vs 0 (0)	..	Frequency of INR testing: 8.14 (4.75) vs 4.47 (3.84); rehospitalization: 3 (1.5%) vs 2 (1.0%); average cost per test: 42.37 (31.60) vs 78.3 (77.91)
Korea^57^	Mobile adherence app	Patient-facing education and adherence–support applications	248 vs 250	6.0	AF patients with stroke risk factors on edoxaban	HF, hypertension, diabetes mellitus, prior stroke/TIA/thromboembolism, vascular disease	DOAC (edoxaban)	65.7 (10.1) vs 65.6 (10.5)	171 (69%) vs 169 (67.6%)	NA (DOAC)	1 (0.4%) vs 1 (0.4%)	0 (0) vs 1 (0.4%); stroke	0 (0) vs 2 (0.8%)	Edoxaban adherence: 147 (73.9%) vs 136 (61.0%)

DTx, digital therapeutics; TTR, time in therapeutic range; AF, artrial fibrillation; DVT, deep thrombosis; PE, pulmonary embolism; TIA, transient ischemic attack; MHVR, mechanical heart valve replacement; MI, myocardial infarction; CD, cardiovascular death; CF, cardiac failure; HF, heart failure; ACS, acute coronary syndrome; SE, systemic embolism; UA, unstable angina; VT, valve thrombosis; AT, atrial thrombosis; SY, systematic thromboembolism; CVR, cardiac valve replacement.

a Intervention group vs. control group

### Intervention Characteristics

Digital therapeutics interventions were delivered via patient-facing smartphone applications (6 RCTs) and internet- or tablet-based telemedicine platforms in clinics (4 RCTs). Across the 10 RCTs, the DTx interventions could be grouped into 3 categories according to their primary therapeutic targets and software design as follows:

1. Warfarin dose-adjustment/INR-guided self-management systems—6 trials^48^^,^^49^^,^^52^, ^53^, ^54^^,^^56^ used internet or mobile platforms connected to portable INR devices. Patients entered INR values and relevant clinical data, using embedded algorithms or rule-based protocols, to generate individualized dosing plans and next-test timing. Clinical oversight and involvement varied, ranging from fully automated expert systems to hybrid models incorporating clinician dashboards for reviewing and overriding dose suggestions. Most systems include educational content, automated reminders, and electronic records.
2. Integrated AF management pathways incorporating anticoagulation—2 trials^50^^,^^55^ implemented broader care pathways with anticoagulation optimization as a core component. IMPACT-AF^55^ provides a clinician-facing decision support system in primary care, influencing prescribing and follow-up without a separate patient application. Conversely, mAFA-II uses a smartphone application linked to a clinician network to implement the ABC pathway, offering both patient and clinician tools.
3. Patient-facing education and adherence applications, such as the Alfalfa smartphone application,^51^ aim to improve anticoagulation (OAC) adherence and knowledge in patients with AF through tailored education, medication reminders, adherence tracking, and remote consultation. The application also allows clinicians to respond to queries and monitor issues, mainly enhancing behavior—knowledge and adherence— because it lacks a dosing algorithm or formal risk stratification. In the ADHERE-App trial,^57^ patients with AF on edoxaban used a smartphone application to confirm daily intake, record missed doses, and receive visual feedback on adherence over time. The internal rules of the application triggered additional prompts or alerts for patients with declining adherence.

Regarding reinforcing support, 1 (10%) trial^55^ supported structured documentation of anticoagulation decisions, and 1 (10%) trial^48^ automatically captured reports into a longitudinal electronic record. Two (20%) Alfalfa-based trials^51^^,^^52^ offered remote consultation, with 1 (10%) trial also incorporating an online anticoagulant community.^51^ None of the studies incorporated passive or automated physiological monitoring and reported machine learning models or adaptive algorithms. All interventions used predefined guideline-based logic. Adherence applications apply simple threshold-based rules to trigger reminders or behavioral feedback. (See intervention details in Supplemental Table 2, available online at https://www.mcpdigitalhealth.org/.)

Overall, the comparison group across 10 RCTs included 2 types: (1) conventional clinic-based anticoagulation or AF care with routine INR monitoring and dose adjustments by health care providers,^48^, ^49^, ^50^, ^51^, ^52^^,^^55^^,^^57^ involving guideline-directed management and education during visits; and (2) self-testing^53^^,^^56^ or self-management,^54^ where patients used portable coagulometers but lacked software-guided dose adjustments or real-time telemedicine. One study used a low-dose INR self-control program,^54^ another had patient self-testing overseen by a clinic,^56^ and a study compared traditional management with an internet-based expert system.^53^

### Meta-analysis of Clinical Outcomes

**Figure 2:**
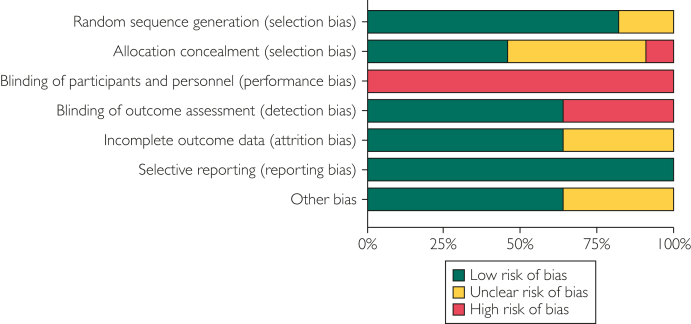
Risk of bias analysis.

Overall, the included studies had a low risk of bias and were of high quality. Bias was most common in participants and personnel blinding, followed by the blinding of outcome assessment. Figure 2 summarizes the risk of bias assessment. The GRADE profiles are presented in Table 2.

**Table 2:** Summary of Primary Outcome

Studies (n)	Certainty assessment					Effect (95% CI)	Certainty
	Risk of bias	Inconsistency	Indirectness	Imprecision	Other considerations		
TTR							
6	Not serious^a^	Serious^b^	Serious^c^	Serious^d^	None	6.94 (−2.82 to 16.70)	⊕⊝⊝⊝ Very low
Major bleeding							
9	Not serious^a^	Not serious	Not serious^e^	Not serious	None	0.47 (0.29-0.76)	⊕⊕⊕⊕ High
Thromboembolic events							
9	Not serious^a^	Not serious	Not serious^e^	Serious^f^	None	0.71 (0.37-1.38)	⊕⊕⊕⊝ Moderate
All-cause death							
7	Not serious^a^	Not serious^g^	Not serious^e^	Serious^f^	None	0.79 (0.34-1.81)	⊕⊕⊕⊝ Moderate

a Several trials were not adequately blinded owing to the nature of the intervention; however, this was unlikely to materially affect objective outcomes.

b Substantial statistical heterogeneity was observed (*I*^2^>75%). Although clinically explainable, the magnitude of heterogeneity remained substantial; therefore, inconsistency was rated as serious.

c One included study used a nonstandard comparator (telephone-assisted self-management) rather than usual care, introducing indirectness. Sensitivity analysis excluding this study resulted in a substantial change in the pooled effect estimate, indicating that this indirectness materially affected the overall result.

d Serious imprecision owing to wide CIs crossing the line of no effect.

e Although 1 trial used a nonstandard comparator, sensitivity analysis excluding this trial yielded consistent results; therefore, no serious concerns about indirectness were identified.

f Serious imprecision owing to wide CIs crossing the null effect and encompassing both benefit and harm.

g Although substantial heterogeneity was observed, inconsistency was not downgraded because it was explained mainly by a single study. Exclusion of this study substantially reduced heterogeneity without materially changing the pooled effect estimate.

**Figure 3:**
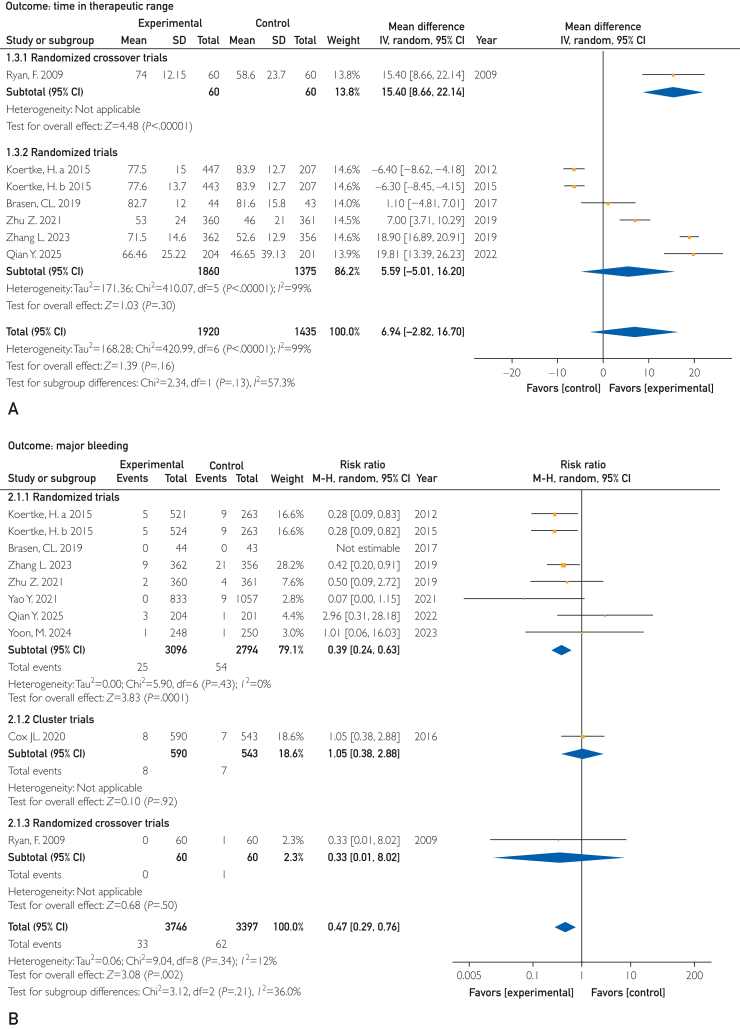

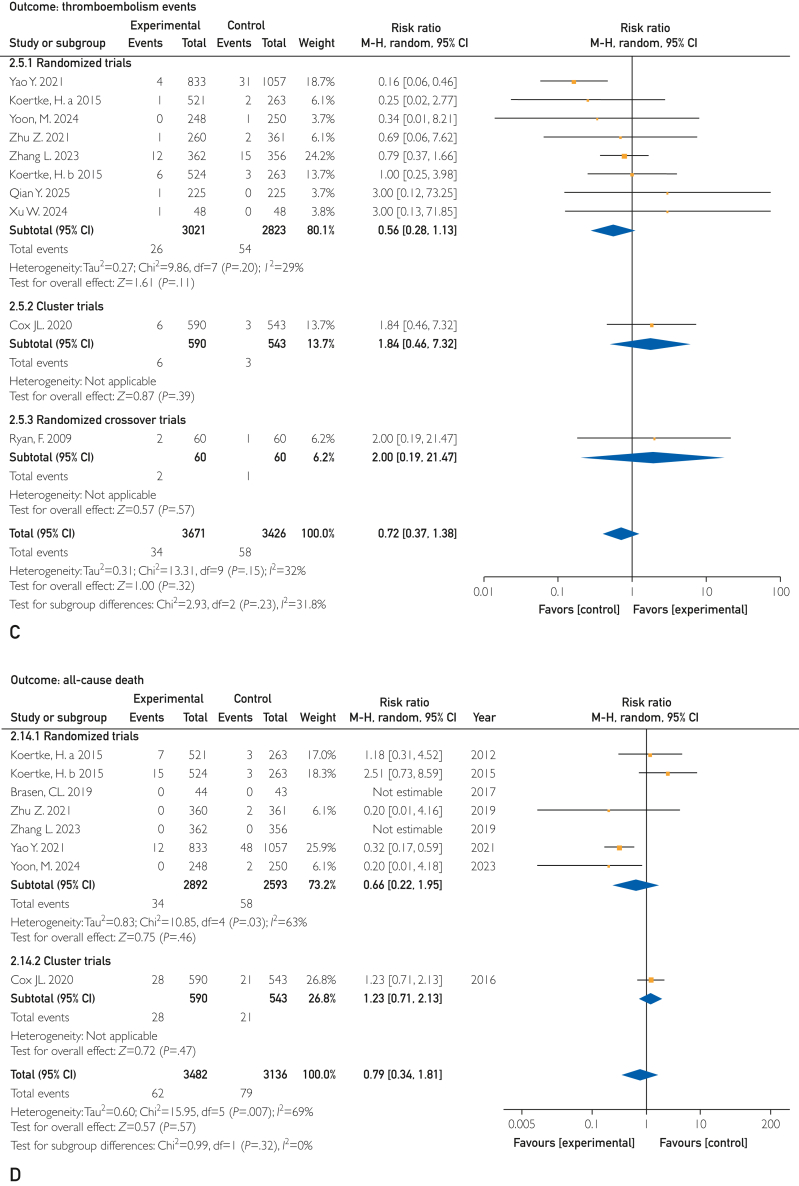
Forest plot for 4 outcomes. (A) Time in therapeutic range. (B) Major bleeding. (C) Thromboembolic events. (D) All-cause death.

Six (60%) studies (n=3355) reported TTR data. Meta-analysis showed that DTx improved TTR compared with usual care (MD, 6.94%; 95% CI, −2.82% to 16.70%; *P*=.16; *I*^2^=99%) (Figure 3A), although this difference was not statistically significant. Quality was graded as very low owing to the wide 95% CI, heterogeneity, and indirectness in the control group by Koertke et al^54^ using telephone-assisted self-management. Sensitivity analysis excluding this study yielded a statistically significant benefit (MD, 12.45%; 95% CI, 5.23%-19.46%; *P<*.001; *I*^2^=93%) (Supplemental Figure 1, available online at https://www.mcpdigitalhealth.org/).

Nine (90%) studies with 7143 participants reported major bleeding values. One study^51^ reported all bleeding events but not major bleeding events. Pooled analysis showed that DTx intervention significantly reduced the incidence of major bleeding compared with usual care (relative risk [RR], 0.47; 95% CI, 0.29-0.76, *P<*.001; *I*^2^=12%) (Figure 3B). The quality of the evidence was ranked high. Although 1 trial used telephone-assisted self-management, we did not downgrade it for indirectness because this approach can be considered an enhancement of standard care.

Thromboembolic events were assessed in 9 (90%) studies involving 7097 patients. The pooled results showed no difference in TEE between the 2 groups (RR, 0.71; 95% CI, 0.37-1.38; *P*=.32; *I*^2^=33%) (Figure 3C). The quality of the evidence was graded as moderate owing to imprecision (wide 95% CI).

Seven (70%) RCTs mentioned all-cause death in 6618 patients. When comparing the DTx intervention with usual care, there were no significant differences in all-cause mortality (RR, 0.79; 95% CI, 0.34-1.81; *P*=.57; *I*^2^=69%) (Figure 3D). The confidence in that estimate was moderate owing to imprecision (wide 95% CI). Although substantial statistical heterogeneity was observed, this heterogeneity was largely attributable to a single study. Exclusion of this study reduced the *I*^2^ to 1%, indicating that the inconsistency was explainable rather than unexplained. Therefore, the certainty of the evidence was not downgraded owing to inconsistency.

Four (40%) studies reported the INR testing frequency. In 2 of these trials, the SDs were not obtained directly or indirectly and were therefore excluded from the analysis. In the remaining 2 trials, the INR testing frequency was defined as the number of INR measurements over the study follow-up. In the 3-month trial, the intervention group underwent 8.14±4.75 vs 4.47±3.84 tests per patient and, in the 12-month trial, 26.0±8.57 vs 20.1±6.47 tests per patient. Pooled analysis showed that DTx interventions were associated with a significantly higher frequency of INR testing (MD, 4.75 tests per patient during follow-up; 95% CI, 2.57-6.94; *P*<.001; *I*^2^=90%) (Supplemental Figure 2, available online at https://www.mcpdigitalhealth.org/). The quality of the evidence was graded as moderate owing to inconsistencies.

Rehospitalization was described in 4 (40%) studies involving 4149 patients. The difference between the groups was not statistically significant (RR, 0.70; 95% CI, 0.32-1.54; *P*=.38; *I*^2^=80%) (Supplemental Figure 3, available online at https://www.mcpdigitalhealth.org/). The quality of the evidence was graded as moderate owing to inconsistencies (important heterogeneity).

Qian et al^52^ assessed the average cost per INR test and cost-effectiveness ratio. Fewer costs were recorded in the intervention group, and the difference was statistically significant (42.37±31.6 vs 78.3±77.91 CNY; *P*<.001). Brasen et al^56^ reported the number of health care professional interactions per month and found fewer interactions in the DTx group.

Three (30%) studies reported medication adherence, and 1 reported incomplete data. The Alfalfa AF trial^48^ showed higher adherence scores in the DTx group, whereas the ADHERE-App trial^57^ reported higher edoxaban adherence (73.9% vs 61.0%).

A sensitivity analysis was performed to exclude the study by Koertke et al,^54^ as previously discussed. After exclusion, the pooled TTR estimate was affected, demonstrating that the DTx intervention significantly improved TTR, whereas the other outcomes remained unchanged. Excluding other individual studies did not significantly affect the pooled estimates for any outcome. Notably, excluding the study by Yao et al^50^ in an all-cause death analysis reduced the *I*^2^ from 69% to 1%. Subgroup analyses were performed for the DTx intervention delivery modality and study population (Supplemental Table 3, available online at https://www.mcpdigitalhealth.org/). Mobile application-based DTx was associated with a lower mortality risk (RR, 0.31; 95% CI, 0.17-0.56; *P*<.001) compared with usual care, whereas we found no difference in mortality between internet system-based DTx and usual care. DTx intervention reduced the risk of major bleeding in patients after cardiac valve replacement; however, the test for subgroup differences was not statistically significant (*P*=.93).

## Discussion

To our knowledge, this is the first systematic review and meta-analysis to synthesize the pooled effectiveness of DTx interventions for OAC management. Previous studies have examined individual components of technology-enabled anticoagulation care, such as self-testing,^15^ telephone services,^13^ online hospitals,^10^ electronic health records, and multitasking telemedicine interventions,^14^ but none have specifically assessed interventions that align with the defining characteristics of DTx or addressed the clinical question of whether software-driven, patient-centered, and evidence-validated digital interventions improve anticoagulation outcomes. To date, we have not identified any DTx prescriptions that have been formally approved for OAC management. Our findings provide the first integrated evidence that DTx-like interventions may hold great promise for the management of OAC therapy.

The primary analysis showed no significant improvement in TTR with DTx; however, a sensitivity analysis excluding a study with telephone-assisted control found a statistically significant benefit. The effects on TEEs and rehospitalization were not significant, but major bleeding was reduced by 53%. Subgroup analysis indicated that mobile application interventions lowered all-cause mortality. DTx also improved testing frequency, medication adherence, and costs.

Digital therapeutics intervention appears superior to technology-unsupported standard care in improving TTR. High heterogeneity in TTR is expected owing to differences in study settings, indications, anticoagulant regimes, and digital intervention designs. Different baseline TTRs may also affect results, with lower baseline TTR populations potentially benefiting more from quality improvement interventions. In our included trials, lower usual care TTR^48^^,^^49^^,^^52^ showed larger gains with DTx support than studies with higher baseline TTR in mature self-management settings.^54^^,^^56^ Outside evidence shows regional TTR variation, from 36% in India to 64% in the United States.^58^ Digital therapeutics may be especially valuable where baseline TTR is low or anticoagulation services are limited.

Although DTx interventions showed trends toward fewer TEEs and rehospitalizations, these were not statistically significant. Low baseline thromboembolism rates in well-anticoagulated populations limit the statistical power unless the sample sizes are large. Rehospitalization is multifactorial, often owing to cardiac disease, multimorbidity, and nonanticoagulation issues; therefore, anticoagulation improvements might only have modest effects during typical trial durations. However, the economic evidence remains insufficient. Only 1 study provided cost data, preventing conclusions regarding cost-effectiveness. With increasing interest in reimbursing DTx, thorough economic evaluations are vital for assessing its value and informing regulatory and reimbursement decisions.

Over the past decade, there have been few large-sample long-term RCTs on anticoagulation management for DTx interventions. This may have led to a misinterpretation of the impact on the outcomes. Validating long-term effects requires not only sustained efficacy but also patient adherence, safety, and disease management. Although the results showed significant control of major bleeding and insignificant effects on thromboembolism and rehospitalization, long-term data to confirm these findings are lacking. In 10 studies, whereas the main clinical indicators were covered, they did not fully reflect patients’ overall, long-term situation in economy, society, psychology, and user experience. Most patients are elderly and may struggle with mobile applications and online data, potentially affecting doctor-patient communication and information transfer. Therefore, multicenter clinical studies with longer follow-up periods are warranted. Future research should involve a variety of disease types and patient groups while addressing practical issues such as network stability, data security, the level of automation, and the need for institutional medication support in the application design to ensure that the findings are broadly applicable and reliable.

The therapeutic effects of DTx interventions are unlikely to result from a single feature but rather from the synergy of several components, including guideline-based and algorithm-driven decision support, structured monitoring and follow-up, behavioral reinforcement and health education, and sustained clinical involvement, which together support safer and more consistent anticoagulation management. In chronic cardiovascular conditions such as AF, mechanical heart valve replacement, and venous thromboembolism, long-term oral anticoagulation management is characterized by substantial workload, complex patient information, high technical content, and a long follow-up period.^54^ Within this context, the role of DTx extends beyond simply enhancing communication or visit scheduling; the software itself can influence dose-adjustment, monitoring intensity, and patient behaviors in a therapeutically meaningful way.

The CDSSs are traditionally designed for clinicians and have proven highly valuable.^59^ In anticoagulation management, however, many patient-facing DTx embedded CDSS-like algorithmic components, such as rule-based dose suggestions, automated alerts for abnormal INR trends, and context-specific self-management guidance to support timely risk detection and structured communication with clinicians. If designed to be accessible, affordable, scalable, and integrated into primary care and community-based services, such interventions that leverage the high reach and time efficiency of mobile technologies and their suitability for multimorbidity management have the potential to integrate more effectively into primary care workflows and support collaborative, knowledge-driven, long-term care. Notably, mobile application-based DTx may be associated with lower mortality than web-based platforms, further suggesting that delivery modality and contextual fit could be important determinants of effectiveness.

Beyond specific functions, the DTx platform can provide a continuous digital interface between patients and health care providers, enabling secure messaging, structured follow-up, health education, and personalized health interventions.^60^ From the patient’s perspective, interaction with health care providers through mobile devices facilitates self-management and, in some settings, improved perceived privacy and convenience. From the clinician’s perspective, DTx tools can serve as auxiliary support systems that help organize information, streamline routine tasks, and allow more time to be devoted to complex decision making. In addition, these platforms generate longitudinal real-world data on anticoagulation trajectories, which can inform future research and iterative refinement of digital algorithms.

Current DTx anticoagulation interventions remain largely dependent on intermittent and manually entered data. None of the included studies reported passive or continuous capture of physiological signals (such as heart rate, blood pressure, or activity) or other sensor-derived data streams. Algorithmically, most systems implemented rule-based logic rather than advanced data-driven models: warfarin self-management platforms relied on dosing algorithms derived from local protocols or guideline-based nomograms; AF integrated-care tools encoded decision trees from national guidelines or the ABC pathway; and adherence-focused applications applied simple threshold-based rules to trigger reminders or feedback. Although this rule-based architecture improves consistency, transparency, and regulatory acceptability compared with ad hoc clinician judgment, it does not yet exploit longitudinal data sets for risk prediction, dynamic individualization of INR targets, or adaptive monitoring intensity.

Looking forward, the increasing availability of wearable devices, home blood pressure monitors, implantable cardiac devices, and smartphones creates opportunities for DTx interventions to incorporate continuous or high-frequency physiological and behavioral data, combined with artificial intelligence–based analytics, to refine risk stratification and tailor monitoring or treatment strategies. Integration with electronic health records could enable automatic retrieval of INR results, renal and hepatic function, and concomitant medications, thereby enhancing safety, personalization, and efficiency in anticoagulation management.^61^ Key challenges include overcoming data silos, ensuring privacy and cybersecurity, and technically embedding algorithms into real-world clinical workflows, particularly in general practice and community settings. In these contexts, medication adherence among culturally and linguistically diverse populations represents an additional and interrelated challenge, shaped by language barriers, limited health literacy, cultural beliefs, and trust in digital systems.^62^^,^^63^ Strengthening collaboration between countries and academic institutions, along with tailored strategies,^2^ robust technical safeguards, and governance frameworks, is essential for DTx in anticoagulation management to evolve from promising experimental tools into reliable components of routine care.

### Limitations

This study had several limitations. This meta-analysis included a relatively small number of studies (n=10), partly because of the strict inclusion criteria. During full-text screening, some articles provided alternative indicators, such as adherence and INR, instead of reporting the primary safety or effectiveness outcomes. Therefore, more narrative reviews are needed to better understand the effectiveness, use patterns, adherence, and practical application of DTx. All included studies were RCTs; however, none were blinded owing to the nature of the intervention, and the level of evidence was moderate. Five of the 10 RCTs were conducted in China and the others in high-income and upper-middle-income countries, which may constrain the generalization of the findings. It is essential for future research to collect evidence in low-income settings, given the known health care equity disparities. The effectiveness of DTx interventions in underserved areas warrants further investigation. The 10 RCTs analyzed included those with diverse DTx designs, underlying conditions, and anticoagulants. However, this reflects the reality of most anticoagulation clinics, and subgroup analyses are required to overcome this limitation. Another limitation is the substantial heterogeneity of TTR, as discussed previously. Finally, most trials had 12 months or less follow-up, limiting the detection of long-term outcomes, such as mortality or rare TEEs. There is also a lack of in-depth economic evaluations.

## Conclusion

Digital therapeutics may contribute to safer and more structured anticoagulation management without demonstrable trade-offs in major clinical outcomes. Future research should focus on adequately powered, longer-term trials with clearly defined DTx mechanisms, consistent comparators, and integrated economic evaluations to better delineate the clinical role of DTx within routine anticoagulation care.

## Potential Competing Interests

Dr You reports institutional grants from The Innovation Project of CAMS (Grant Number: 2021-I2M-1-046. Dr Kar reports institutional grants from UK NIHR Clinical Lecturer award (Grant Number: CL-001-2023-24). Dr Jonnagaddala reports institutional grants from the Australian National Health and Medical Research Council (Grant Number: GNT1192469 and GNT2038330), Research Technology Services at UNSW Sydney, Google Cloud Research (Award Number: GCP19980904), NVIDIA Academic Hardware grant programs, and 2025 IGD GRIP Funding; royalties from Elsevier; consulting fees from UNICEF Lebanon, CARE PNG, and WHO; honoraria from Ministry of Health, Jakarta, Indonesia; is the editorial board member of *NPJ Precision Oncology*; and is the guest editor of *NPJ Digital Medicine*. The authors report no competing interests.
