# Supplementary material for: The Effectiveness of Digital Therapeutics Intervention in Oral Anticoagulation Management: A Systematic Review and Meta-analysis
Source: Mayo Clin Proc Digit Health. 2026 Jan 20;4(1):100336. doi: 10.1016/j.mcpdig.2026.100336 (PMC12937170; doi:10.1016/j.mcpdig.2026.100336)
Supplement: Supplementary Material [file mmc1.docx]

**Supplementary Material**

**Supplemental Table 1. PRISMA 2020 checklist and location of reported items**

| **Section and Topic** | **Item #** | **Checklist item** | **Location where item is reported** |
| --- | --- | --- | --- |
| **TITLE** | | |  |
| Title | 1 | Identify the report as a systematic review. | Page 1 |
| **ABSTRACT** | | |  |
| Abstract | 2 | See the PRISMA 2020 for Abstracts checklist. | Page 3 |
| **INTRODUCTION** | | |  |
| Rationale | 3 | Describe the rationale for the review in the context of existing knowledge. | Page 5,6 |
| Objectives | 4 | Provide an explicit statement of the objective(s) or question(s) the review addresses. | Page 6 |
| **METHODS** | | |  |
| Eligibility criteria | 5 | Specify the inclusion and exclusion criteria for the review and how studies were grouped for the syntheses. | Page 8,9 |
| Information sources | 6 | Specify all databases, registers, websites, organisations, reference lists and other sources searched or consulted to identify studies. Specify the date when each source was last searched or consulted. | Page 7 |
| Search strategy | 7 | Present the full search strategies for all databases, registers and websites, including any filters and limits used. | Supplementary 2 |
| Selection process | 8 | Specify the methods used to decide whether a study met the inclusion criteria of the review, including how many reviewers screened each record and each report retrieved, whether they worked independently, and if applicable, details of automation tools used in the process. | Page 7 |
| Data collection process | 9 | Specify the methods used to collect data from reports, including how many reviewers collected data from each report, whether they worked independently, any processes for obtaining or confirming data from study investigators, and if applicable, details of automation tools used in the process. | Page 9 |
| Data items | 10a | List and define all outcomes for which data were sought. Specify whether all results that were compatible with each outcome domain in each study were sought (e.g. for all measures, time points, analyses), and if not, the methods used to decide which results to collect. | Page 9 |
|  | 10b | List and define all other variables for which data were sought (e.g. participant and intervention characteristics, funding sources). Describe any assumptions made about any missing or unclear information. | Page 9 |
| Study risk of bias assessment | 11 | Specify the methods used to assess risk of bias in the included studies, including details of the tool(s) used, how many reviewers assessed each study and whether they worked independently, and if applicable, details of automation tools used in the process. | Page 9 |
| Effect measures | 12 | Specify for each outcome the effect measure(s) (e.g. risk ratio, mean difference) used in the synthesis or presentation of results. | Page 10 |
| Synthesis methods | 13a | Describe the processes used to decide which studies were eligible for each synthesis (e.g. tabulating the study intervention characteristics and comparing against the planned groups for each synthesis (item #5)). | Page 10 |
|  | 13b | Describe any methods required to prepare the data for presentation or synthesis, such as handling of missing summary statistics, or data conversions. | Page 14 |
|  | 13c | Describe any methods used to tabulate or visually display results of individual studies and syntheses. | Page 10 |
|  | 13d | Describe any methods used to synthesize results and provide a rationale for the choice(s). If meta-analysis was performed, describe the model(s), method(s) to identify the presence and extent of statistical heterogeneity, and software package(s) used. | Page 10 |
|  | 13e | Describe any methods used to explore possible causes of heterogeneity among study results (e.g. subgroup analysis, meta-regression). | Page 10 |
|  | 13f | Describe any sensitivity analyses conducted to assess robustness of the synthesized results. | Page 10 |
| Reporting bias assessment | 14 | Describe any methods used to assess risk of bias due to missing results in a synthesis (arising from reporting biases). | Page 10 |
| Certainty assessment | 15 | Describe any methods used to assess certainty (or confidence) in the body of evidence for an outcome. | Page 9 |
| **RESULTS** | | |  |
| Study selection | 16a | Describe the results of the search and selection process, from the number of records identified in the search to the number of studies included in the review, ideally using a flow diagram. | Page 10  Figure 1 |
|  | 16b | Cite studies that might appear to meet the inclusion criteria, but which were excluded, and explain why they were excluded. | Not Mentioned |
| Study characteristics | 17 | Cite each included study and present its characteristics. | Page 10,11  Table 1 |
| Risk of bias in studies | 18 | Present assessments of risk of bias for each included study. | Page 12  Figure 2 |
| Results of individual studies | 19 | For all outcomes, present, for each study: (a) summary statistics for each group (where appropriate) and (b) an effect estimate and its precision (e.g. confidence/credible interval), ideally using structured tables or plots. | Table 1  Figure 3 |
| Results of syntheses | 20a | For each synthesis, briefly summarise the characteristics and risk of bias among contributing studies. | Page 12-16 |
|  | 20b | Present results of all statistical syntheses conducted. If meta-analysis was done, present for each the summary estimate and its precision (e.g. confidence/credible interval) and measures of statistical heterogeneity. If comparing groups, describe the direction of the effect. | Page 12-16  Figure 3  Supplementary 3 |
|  | 20c | Present results of all investigations of possible causes of heterogeneity among study results. | Page 15 |
|  | 20d | Present results of all sensitivity analyses conducted to assess the robustness of the synthesized results. | Page 14 |
| Reporting biases | 21 | Present assessments of risk of bias due to missing results (arising from reporting biases) for each synthesis assessed. | Page 9 |
| Certainty of evidence | 22 | Present assessments of certainty (or confidence) in the body of evidence for each outcome assessed. | Table 2 |
| **DISCUSSION** | | |  |
| Discussion | 23a | Provide a general interpretation of the results in the context of other evidence. | Page 15,16 |
|  | 23b | Discuss any limitations of the evidence included in the review. | Page 19,20 |
|  | 23c | Discuss any limitations of the review processes used. | Page 19,20 |
|  | 23d | Discuss implications of the results for practice, policy, and future research. | Page 17,18 |
| **OTHER INFORMATION** | | |  |
| Registration and protocol | 24a | Provide registration information for the review, including register name and registration number, or state that the review was not registered. | Page 7 |
|  | 24b | Indicate where the review protocol can be accessed, or state that a protocol was not prepared. | Page 7 |
|  | 24c | Describe and explain any amendments to information provided at registration or in the protocol. | Not Mentioned |
| Support | 25 | Describe sources of financial or non-financial support for the review, and the role of the funders or sponsors in the review. | Page 2 |
| Competing interests | 26 | Declare any competing interests of review authors. | Page 2 |
| Availability of data, code and other materials | 27 | Report which of the following are publicly available and where they can be found: template data collection forms; data extracted from included studies; data used for all analyses; analytic code; any other materials used in the review. | Page 21 |

**Supplemental Methods
Search strategy**

PubMed

Search data: June 20, 2025

(("Telemedicine"[Mesh] OR Telemedicine[Title/Abstract] OR mhealth[Title/Abstract] OR "mobile health"[Title/Abstract] OR "digital health"[Title/Abstract] OR "Smartphone App*"[Title/Abstract] OR "Smartphone application"[Title/Abstract] OR "APP, Mobile*"[Title/Abstract] OR eHealth[Title/Abstract] OR Telecommunication*[Title/Abstract] OR telemonitoring[Title/Abstract] OR "digital therapy*"[Title/Abstract])) AND ((("Anticoagulants"[Mesh] OR Anticoagulants[Title/Abstract] OR "Heparin"[Mesh] OR Heparin*[Title/Abstract] OR "Warfarin"[Mesh] OR Warfarin*[Title/Abstract] OR DOAC*[Title/Abstract] OR DOAC*[Title/Abstract] OR "New Oral Anticoagulant*"[Title/Abstract] OR Rivaroxaban[Title/Abstract] OR Apixaban[Title/Abstract] OR Dabigatran*[Title/Abstract] OR Edoxaban[Title/Abstract] OR Betrixaban[Title/Abstract] OR "Blood Thinner*"[Title/Abstract] OR "Anticoagulant Agent*"[Title/Abstract] OR "Antithrombotic Agent*"[Title/Abstract] OR anticoagulation[Title/Abstract] OR "Anticoagulation Therapy*"[Title/Abstract] OR "Anticoagulation Agents"[Title/Abstract] OR "Agents, Anticoagulation"[Title/Abstract] OR "Anticoagulant Drugs"[Title/Abstract] OR "Drugs, Anticoagulant"[Title/Abstract] OR "Indirect Thrombin Inhibitors"[Title/Abstract] OR "Thrombin Inhibitors, Indirect"[Title/Abstract] OR antithrombotics[Title/Abstract] OR "anticoagulation treatment"[Title/Abstract] OR "anticoagulation management"[Title/Abstract] OR "anticoagulation services"[Title/Abstract])) OR (("Venous Thromboembolism"[Mesh] OR Venous Thromboembolism*[Title/Abstract] OR VTE[Title/Abstract] OR Deep Vein Thrombosis*[Title/Abstract] OR Phlebothrombosis*[Title/Abstract] OR "Pulmonary Embolism"[Mesh] OR Pulmonary Embolism*[Title/Abstract] OR PE[Title/Abstract] OR "Lung Emboli*"[Title/Abstract] OR "Venous Thrombosis"[Mesh] OR Venous Thrombosis*[Title/Abstract] OR "Venous Thromboembolic Disease*"[Title/Abstract])) OR (("Atrial Fibrillation"[Mesh] OR ("atrial fibrill*"[Title/Abstract] OR "auricular fibrill*"[Title/Abstract] OR AFib[Title/Abstract] OR AF[Title/Abstract] OR "A Fib"[Title/Abstract]))))

Web of Science

Search data: June 20, 2025

(TS=(Telemedicine) OR AB= (Telemedcine OR mhealth OR mobile health OR digital health OR telemonitoring OR “Smartphone App?” OR Smartphone application OR “APP?, Mobile” OR eHealth OR Telecommunication OR Computer-Assisted Drug therapy OR “digital therap*”)) AND ((TS=("Anticoagulants") OR AB=(Anticoagulant* OR Heparin* OR Warfarin* OR DOAC* OR DOAC* OR "New Oral Anticoagulant*" OR Rivaroxaban OR Apixaban OR Dabigatran* OR Edoxaban OR Betrixaban OR "Blood Thinner*" OR "Anticoagulant Agent*" OR "Antithrombotic Agent*" OR anticoagulation OR "Anticoagulation Therapy*" OR "Anticoagulation Agents" OR "Agents, Anticoagulation" OR "Anticoagulant Drugs" OR "Drugs, Anticoagulant" OR "Indirect Thrombin Inhibitors" OR "Thrombin Inhibitors, Indirect" OR antithrombotics OR "anticoagulation treatment" OR "anticoagulation management" OR "anticoagulation services")) OR (TS=("Venous Thromboembolism") OR AB=(Venous Thromboembolism* OR VTE OR Deep Vein Thrombosis* OR Phlebothrombosis* OR "Pulmonary Embolism" OR Pulmonary Embolism* OR PE OR "Lung Emboli*" OR "Venous Thrombosis" OR Venous Thrombosis* OR "Venous Thromboembolic Disease*")) OR (TS=("Atrial Fibrillation") OR AB=("atrial fibrill*" OR "auricular fibrill*" OR AFib OR AF OR "A Fib")))

Cochrane

Search data: June 20, 2025

#1 MeSH descriptor: [Anticoagulants] explode all trees

#2 MeSH descriptor: [Heparin] explode all trees

#3 MeSH descriptor: [Warfarin] explode all trees

#4 (anticoagulants OR heparin* OR warfarin* OR DOAC* OR DOAC* OR (new NEXT oral NEXT anticoagulant*) OR rivaroxaban OR apixaban OR dabigatran* OR edoxaban OR betrixaban OR (blood NEXT thinner*) OR (anticoagulant NEXT agent*) OR (antithrombotic NEXT agent*) OR anticoagulation OR (anticoagulation NEXT therapy*) OR "anticoagulation agents OR "agents, anticoagulation" OR "anticoagulant drugs" OR "drugs, anticoagulant" OR "indirect thrombin inhibitors" OR "thrombin inhibitors, indirect" OR antithrombotics OR "anticoagulation treatment" OR "anticoagulation management" OR "anticoagulation services"):ti,ab,kw

#5 MeSH descriptor: [Venous Thromboembolism] explode all trees

#6 MeSH descriptor: [Deep Vein Thrombosis] explode all trees

#7 MeSH descriptor: [Pulmonary Embolism] explode all trees

#8 MeSH descriptor: [Venous Thrombosis] explode all trees

#9 ((venous NEXT thromboembolism*) OR VTE OR (deep NEXT vein NEXT thrombosis*) OR phlebothrombosis* OR (pulmonary NEXT embolism*) OR PE OR (lung NEXT emboli*) OR (venous NEXT thrombosis*)):ti,ab,kw

#10 MeSH descriptor: [Atrial Fibrillation] explode all trees

#11 (atrial NEXT fibrill* OR auricular NEXT fibrill* OR AFib OR AF OR "A Fib"):ti,ab,kw

#12 #1 OR #2 OR #3 OR #4 OR #5 OR #6 OR #7 OR #8 OR #9 OR #10 OR #11

#13 MeSH descriptor: [Telemedicine] explode all trees

#14 (telemedicine OR mhealth OR "mobile health" OR "digital health" OR (smartphone NEXT app*) OR "smartphone application" OR (app, NEXT mobile*) OR eHealth OR telecommunication* OR telemonitoring OR (digital NEXT therap*)):ti,ab,kw

#15 #13 OR #14

#16 #12 AND #15

Embase

Search data: June 20, 2025

(('telemedicine'/exp OR 'telemedicine':ab,ti OR 'mhealth':ab,ti OR 'mobile health':ab,ti OR 'digital health':ab,ti OR 'Smartphone App*':ab,ti OR 'Smartphone application':ab,ti OR 'APP, Mobile*':ab,ti OR 'eHealth':ab,ti OR 'Telecommunication*':ab,ti OR 'telemonitoring':ab,ti OR 'digital therapy*':ab,ti)) AND ((('anticoagulant agent'/exp OR 'Anticoagulants':ab,ti OR 'heparin'/exp OR 'Heparin*':ab,ti OR 'warfarin'/exp OR 'Warfarin*':ab,ti OR 'DOAC*':ab,ti OR 'DOAC*':ab,ti OR 'New Oral Anticoagulant*':ab,ti OR 'Rivaroxaban':ab,ti OR 'Apixaban':ab,ti OR 'Dabigatran*':ab,ti OR 'Edoxaban':ab,ti OR 'Betrixaban':ab,ti OR 'Blood Thinner*':ab,ti OR 'Anticoagulant Agent*':ab,ti OR 'Antithrombotic Agent*':ab,ti OR 'anticoagulation':ab,ti OR 'Anticoagulation Therapy*':ab,ti OR 'Anticoagulation Agents':ab,ti OR 'Agents, Anticoagulation':ab,ti OR 'Anticoagulant Drugs':ab,ti OR 'Drugs, Anticoagulant':ab,ti OR 'Indirect Thrombin Inhibitors':ab,ti OR 'Thrombin Inhibitors, Indirect':ab,ti OR 'antithrombotics':ab,ti OR 'anticoagulation treatment':ab,ti OR 'anticoagulation management':ab,ti OR 'anticoagulation services':ab,ti)) OR (('vein thrombosis'/exp OR 'Venous Thromboembolism*':ab,ti OR 'VTE':ab,ti OR 'Deep Vein Thrombosis*':ab,ti OR 'Phlebothrombosis*':ab,ti OR 'lung embolism'/exp OR 'Pulmonary Embolism*':ab,ti OR 'PE':ab,ti OR 'Lung Emboli*':ab,ti OR 'vein thrombosis'/exp OR 'Venous Thrombosis*':ab,ti OR 'Venous Thromboembolic Disease*':ab,ti)) OR (('atrium fibrillation'/exp OR ('atrial fibrill*':ab,ti OR 'auricular fibrill*':ab,ti OR 'AFib':ab,ti OR 'AF':ab,ti OR 'A Fib':ab,ti)

## Supplemental Table 2. Characteristics of interventions

| **Study_ID** | **Portable Coagulometers** | **Web Platform** | **Remote Monitoring** | **Automated Alerts** | **Mobile Apps** | **Software Driven** | **User DataInput** | **Algorithm Embedded** | **Physician Involvement** | **Anticoagulation Management** | **Disease Management** | **Health Education** | **Reinforcing Support** |
| --- | --- | --- | --- | --- | --- | --- | --- | --- | --- | --- | --- | --- | --- |
| 40_Zhu_2021 | No – INR measured in local hospitals/labs; home point-of-care coagulometers not used. | Yes – internet-based warfarin management platform used for data upload and storage. | Yes – clinicians remotely review uploaded INR and laboratory results to guide therapy. | Unclear – platform likely includes scheduling or contact features, but automated alerts (SMS/push) are not explicitly described. | Yes – follow-up software is complemented by patient-facing mobile apps. | Yes – software platform is the central vehicle for delivering the intervention. | Yes – patients input INR values and other clinical data into the system or app. | Partial – dose adjustment follows guideline-based INR targets and local protocols, but specific algorithmic rules are not fully detailed. | Yes – physicians/clinical team review transmitted data and provide management decisions. | Yes – primary focus is long-term warfarin management (INR/TTR and complications) after MHVR. | No – broader multimorbidity or integrated chronic disease management is not systematically described. | Yes – anticoagulation education and app-based information are provided to patients. | Yes – structured follow-up, remote feedback and ongoing digital contact reinforce adherence and engagement. |
| 41_Zhang_2023 | Unclear – patients report INR results, but routine use of home coagulometers is not explicitly stated. | Yes – social app is supported by an online platform for data transfer and storage. | Yes – clinicians monitor INR and anticoagulation status remotely through the app between clinic visits. | Partial – interaction and reminders through the app are described, but specific automated alert modalities (e.g., SMS vs in-app) are not clearly reported. | Yes – the intervention is delivered via a patient-facing smartphone social application. | Yes – the social app is the central tool for remote anticoagulation management. | Yes – patients enter INR values, dosing information and relevant clinical data into the app. | Partial – management appears to follow predefined INR targets and structured rules; detailed algorithmic logic is not fully reported. | Yes – healthcare providers review app data and give dosage and follow-up advice. | Yes – main aim is to improve warfarin control (e.g., TTR) after MHVR. | No – the intervention does not describe a comprehensive multimorbidity or AF pathway beyond anticoagulation. | Yes – the app provides anticoagulation-related education and medication guidance. | Yes – interactive communication, reminders and potential community features provide reinforcing social and behavioural support. |
| 42_Yao_2021_mAFAII | No – anticoagulation is monitored through usual INR pathways; home coagulometers are not part of the intervention. | Yes – the mHealth-supported pathway is backed by a central digital platform for data management. | Yes – AF status, treatment and risk factors are followed remotely via the mHealth integrated care model. | Yes – the app issues reminders and prompts linked to ABC pathway goals and follow-up schedules. | Yes – a dedicated smartphone mHealth application is used by patients and clinicians. | Yes – the mHealth platform is the main vehicle for implementing the ABC integrated care pathway. | Yes – structured clinical data (symptoms, comorbidities, blood pressure, medications) are entered by clinicians and/or patients. | Yes – clinical decision logic encodes ABC pathway and guideline recommendations for AF and anticoagulation management. | Yes – clinicians actively use the system to adjust therapy and manage multimorbidity. | Yes – optimisation of oral anticoagulant use is a key component of the intervention. | Yes – broader AF disease management, including symptom control and comorbidity/risk-factor management, is explicitly targeted. | Yes – patients receive structured education on AF, anticoagulation and lifestyle/risk-factor control via the programme. | Yes – follow-up prompts, pathway-based reminders and team-based management provide strong reinforcing support. |
| 43_Xu_2024_Alfalfa_AF | No – INR is generally tested in clinics or hospitals; home INR self-testing devices are not described. | Yes – Alfalfa runs on a digital backend, although the article emphasises the smartphone front end. | Yes – patients can report status and receive management remotely through the app between visits. | Yes – the app provides medication reminders and follow-up notifications to support regular anticoagulation use. | Yes – Alfalfa is a patient-facing smartphone application used for AF patients on OAC. | Yes – the app is the central digital module delivering the intervention. | Yes – patients enter medication use, symptoms and other relevant information into the app. | Partial – rule-based reminders and some decision-support functions align with guidelines, but detailed algorithmic models are not reported. | Yes – clinicians provide remote consultation and respond to patient queries through the app. | Yes – intervention aims to improve anticoagulation knowledge, adherence and satisfaction among AF patients. | Partial – some broader AF management elements are present, but the main focus is anticoagulation rather than a fully integrated pathway. | Yes – explicit health education modules regarding AF and anticoagulation are part of the app. | Yes – reminders, consultation access and interactive features act as reinforcing support mechanisms. |
| 44_Qian_2025_Alfalfa_CVR | Unclear – INR testing is likely performed in health facilities; home coagulometer use is not clearly specified. | Yes – the “Internet + pharmacy care” model combines an online platform with the Alfalfa app for data and service flow. | Yes – pharmacists remotely monitor warfarin therapy based on INR and medication data transmitted through the system. | Yes – the intervention includes automated reminders for INR testing, follow-up and possibly safety alerts, although detailed technical implementation is not fully described. | Yes – the Alfalfa smartphone app functions as the main patient interface. | Yes – the internet platform and app together drive the intervention and pharmacy-based follow-up. | Yes – patients and pharmacists enter INR values, dosing, concomitant medications and other relevant clinical information. | Partial – guideline-based pharmacy rules and interaction checks are applied; more advanced predictive or adaptive models are not reported. | Yes – pharmacists (and collaborating clinicians) actively review data and provide therapeutic recommendations. | Yes – main goal is to improve warfarin control (TTR, complications, costs) in patients after cardiac valve replacement. | Partial – the intervention focuses on post-valve replacement care but does not implement a comprehensive multimorbidity pathway. | Yes – pharmaceutical counselling and education on warfarin therapy are important components. | Yes – frequent follow-up, automatic reminders and bidirectional communication constitute reinforcing support. |
| 45_Ryan_2009 | Yes – patients perform home INR self-testing using a portable point-of-care device. | Yes – an internet-based expert system (web page) is used for data submission and feedback. | Yes – anticoagulation is supervised at a distance, with patients managed via the expert system and the anticoagulation service. | Partial – the system automatically returns dose and next test date on screen; specific use of SMS or phone alerts is not reported. | No – the intervention is delivered via a web interface rather than a dedicated mobile app. | Yes – the expert system software is the core component of the intervention model. | Yes – patients enter INR values and treatment-related data into the web system. | Yes – embedded dosing algorithms calculate warfarin dose and testing intervals from predefined rules. | Yes – the anticoagulation management service oversees patients and can override algorithmic recommendations. | Yes – primary focus is to improve warfarin control through supervised self-testing and algorithm-guided dose adjustment. | No – there is no explicit, structured management of comorbid conditions beyond anticoagulation. | Limited – patients receive initial training; extensive ongoing educational modules are not a central feature. | Yes – increased testing frequency, immediate feedback and structured self-management reinforce adherence and safety. |
| 46_Koertke_2015 | Yes – patients use point-of-care INR devices at home for very low-dose INR self-control. | Yes – a telemedicine system is used for data transfer and management of self-control results. | Yes – the study is explicitly described as telemedicine-guided INR self-control with remote supervision. | Partial – predefined criteria for INR values and dose changes trigger interactions; exact alert modality (e.g., SMS vs call) is not fully specified. | No – the intervention relies on telemedicine software, not on a smartphone app. | Yes – telemedicine software and associated infrastructure are central to the intervention. | Yes – patients report INR and dosing information into the system for assessment. | Yes – target ranges and criteria (e.g., INR <1·8 or >4·5, dose-change thresholds) are encoded as an explicit rule set. | Yes – specialist teams monitor data and intervene according to these criteria-based rules. | Yes – intervention is designed to optimise safe, effective warfarin therapy in mechanical heart valve patients. | No – the programme does not describe broader chronic disease management beyond anticoagulation. | Limited – initial education and training are provided; ongoing telemedicine-based education content is not the main focus. | Yes – structured self-testing, criteria-based contacts and remote feedback provide reinforcing support. |
| 47_Cox_2020_IMPACT_AF | No – INR testing follows usual laboratory processes; home coagulometers are not used. | Yes – a computerized clinical decision support tool runs on primary care computers/servers. | No – the tool is used at the point of care during visits rather than for continuous remote monitoring. | Yes – the CDSS provides prompts and reminders when patients meet guideline criteria or have care gaps. | No – the intervention is clinician-facing, delivered on desktop/tablet rather than via a patient app. | Yes – the CDSS software is the core intervention in this cluster RCT. | Yes – clinicians input or retrieve structured patient data (AF status, comorbidities, therapies) into/from the tool. | Yes – guideline-based algorithms and ABC pathway decision trees are embedded in the software. | Yes – primary care physicians use the tool to guide anticoagulation decisions and broader AF management. | Yes – optimisation of oral anticoagulant initiation and maintenance is one of the tool’s main aims. | Yes – integrated AF management (stroke prevention, symptom control, comorbidity and risk-factor management) is targeted. | Partial – the system may prompt education/counselling, but patient-facing educational modules are not the primary feature. | Yes – by standardising decisions and follow-up planning, the tool reinforces consistent management and action on identified gaps. |
| 48_Brasen_2019 | Yes – patients use CoaguChek XS portable INR devices at home for self-testing. | Yes – CSO/AC telemedicine software is used to store data and manage patient–provider interactions. | Yes – a real-time supervised telemedicine solution provides continuous remote oversight of warfarin therapy. | Partial – predefined INR thresholds, dose-change criteria and missing data trigger interactions; specific alert channels are not fully described. | No – technology is telemedicine software accessed from home rather than a smartphone app. | Yes – CSO/AC software is the central component enabling supervised patient self-management. | Yes – patients submit INR values and dosing information, which are captured in the system. | Yes – explicit criteria (INR limits, warfarin/week change, missing values) function as embedded decision logic. | Yes – healthcare professionals monitor patients and respond when criteria are met. | Yes – core aim is to support home warfarin management and maintain stable INR control. | No – intervention does not formally address comorbidities or broader chronic disease pathways. | Limited – some instruction and ongoing information are provided; structured educational modules are not central. | Yes – targeted, criteria-based interactions, combined with regular self-testing, reinforce safe and adherent behaviour. |
| 49_Yoon_2024_ADHERE_App | No – NOAC therapy (edoxaban) is used, and INR monitoring is not required. | Yes – the app is supported by a backend system and clinical data infrastructure, although the publication emphasises the smartphone interface. | Yes – clinicians can review adherence summaries remotely, but no physiological monitoring is conducted. | Yes – the app sends push notifications reminding patients to take edoxaban and, in some cases, to measure blood pressure or heart rate. | Yes – ADHERE-App is a patient-facing smartphone adherence application. | Yes – the app is the main digital component responsible for delivering the adherence intervention. | Yes – patients enter medication intake and, in some cases, self-measured vital signs into the app. | Partial – rule-based thresholds (e.g., ≥80 % days covered) structure feedback, but there are no advanced predictive models described. | Yes – clinicians access adherence data during follow-up and can modify management accordingly. | Yes – intervention explicitly targets improvement in adherence to oral anticoagulation (edoxaban). | No – broader multimorbidity or AF pathway management is not systematically embedded into the app. | Yes – the app provides information on edoxaban use and AF-related risk, though depth of educational content is modest. | Yes – frequent reminders, feedback on adherence and visualisation of dosing history provide strong reinforcing support. |

**Supplemental Figure 1. Forest plot of sensitivity analysis for time in therapeutic range**


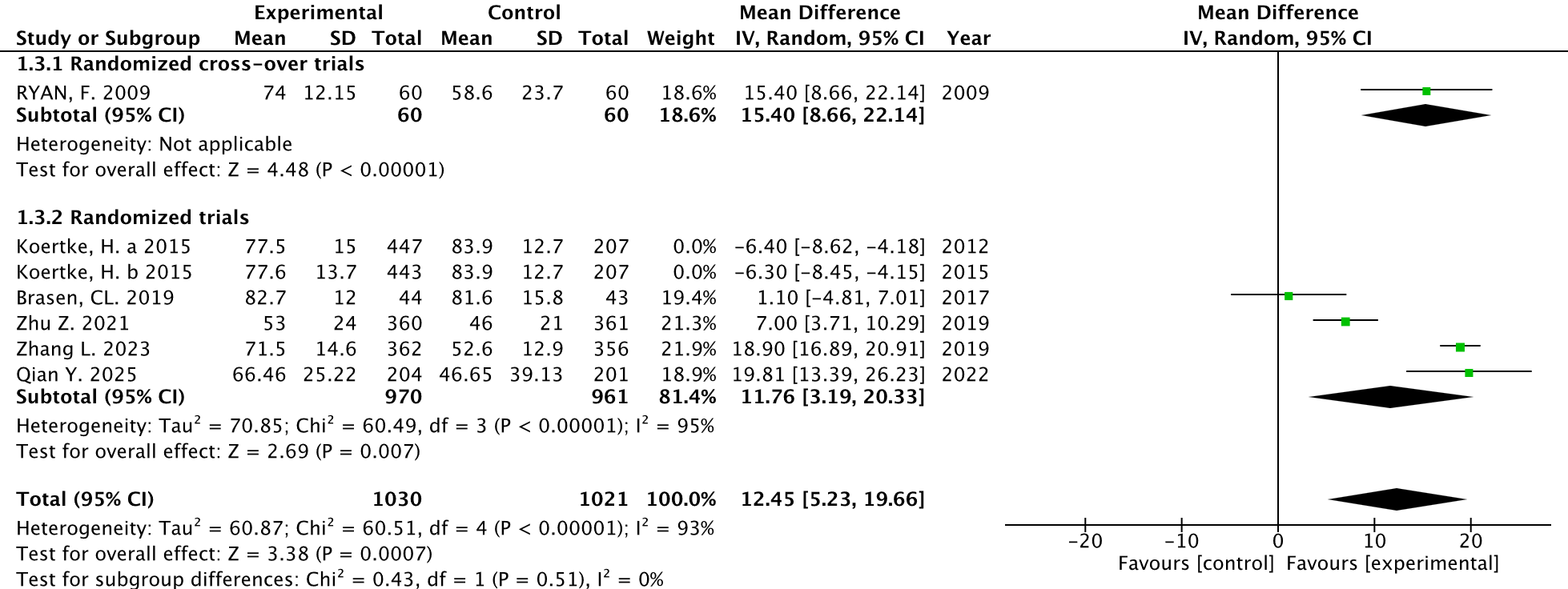


Forest plot showing the sensitivity analysis for time in therapeutic range after excluding the study with a telephone-assisted self-management comparator.

**Supplemental Figure 2. Forest plot of INR testing frequency**


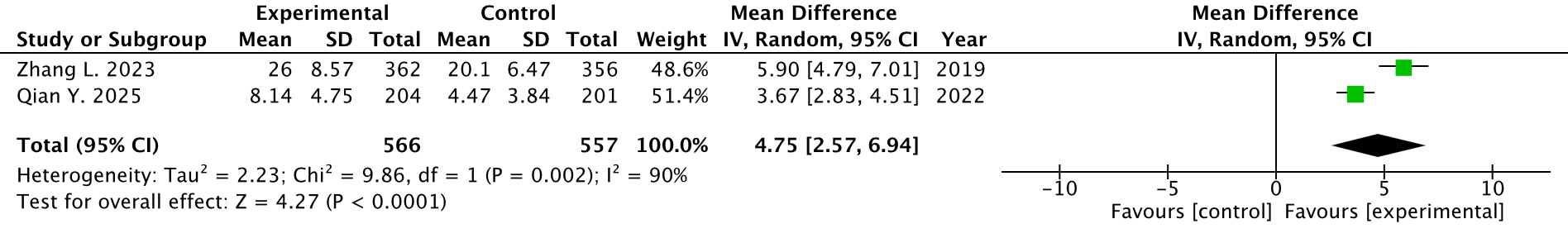


**Supplemental Figure 3. Forest plot of rehospitalization**


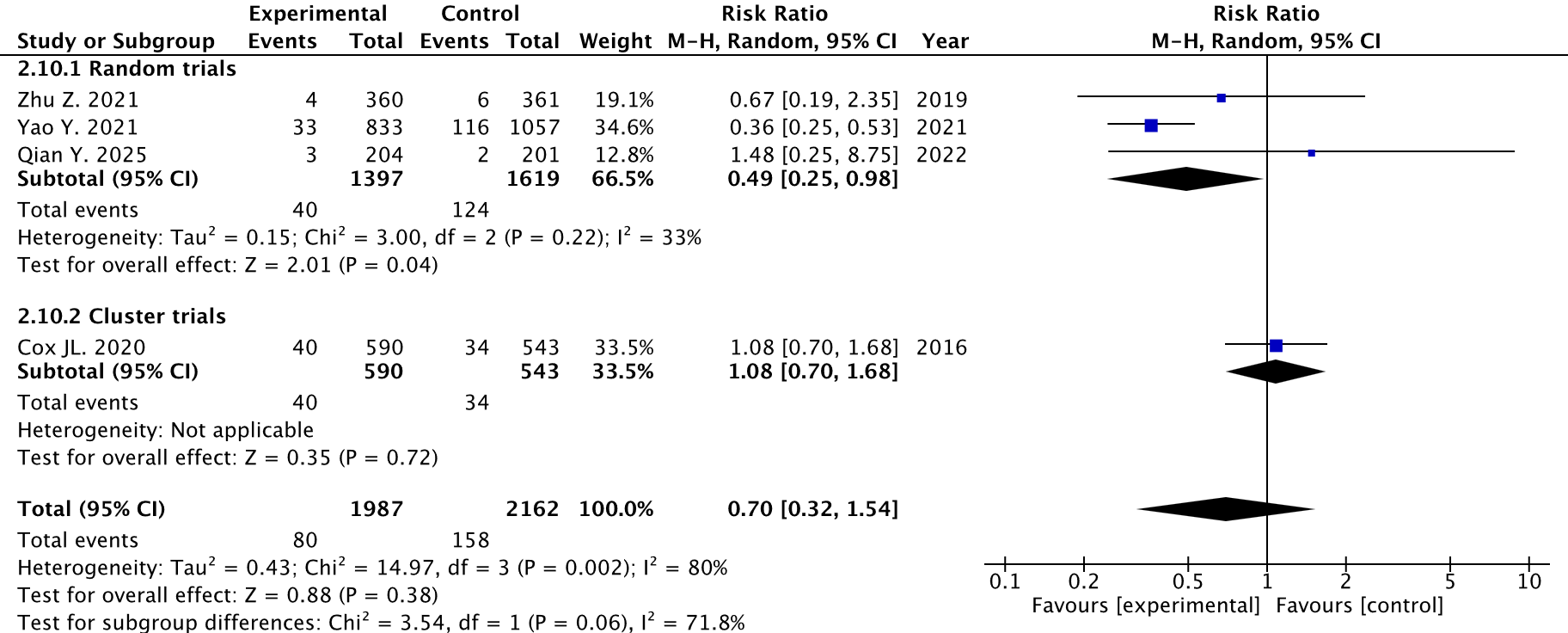


**Supplemental Table 3. Subgroup analyses of randomized controlled trials by intervention modality and patient population.**

| Category | Outcome | | | |
| --- | --- | --- | --- | --- |
|  | TTR^a^, MD^b^, (95% CI) | Major bleeding, RR^c^ (95% CI) | Total thromboembolic events, RR^c^ (95%CI) | All-cause death, RR^c^ (95%CI) |
| DTx intervention delivery modality | | | | |
| Mobile app | 11.68 (3.22, 20.14) | 0.53 (0.28, 1.02) | 0.84 (0.43, 1.64) | 0.31 (0.17, 0.56) |
| Internet-based systems | -0.18 (-7.62, 7.25) | 0.28 (0.13, 0.59) | 1.16 (0.50, 2.69) | 1.36 (0.85, 2.18) |
| Single tablet-software | 1.10 (-4.81, 7.01) | Not estimable | No values | Not estimable |
|  | *P* value for subgroup | | | |
|  | 0.07 | 0.21 | 0.56 | 0.0001 |
| Patient Population | | | | |
| AF^d^ patients | No values | 0.55 (0.10, 2.99) | 0.64 (0.12, 3.36) | 1.40 (0.51, 3.87) |
| Patients after CVR^e^ | 6.47 (-5.45, 18.40) | 0.40 (0.24, 0.65) | 0.79 (0.43, 1.47) | 0.55 (0.17, 1.82) |
| General patients with warfarin therapy | 5.30 (-5.03, 15.63) | 0.33 (0.01, 8.02) | 2.03 (0.18, 23.06) | Not estimable |
|  | *P* value for subgroup | | | |
|  | 0.88 | 0.93 | 0.73 | 0.24 |

^a^TTR: time in therapeutic range

^b^MD: mean difference

^c^RR: relative risk

^d^AF: Atrial fibrillation

^e^CVR: cardiac valve replacement
