# Supplementary material for: The Effectiveness of Digital Therapeutics Intervention in Oral Anticoagulation Management: A Systematic Review and Meta-analysis
Source: Mayo Clin Proc Digit Health. 2026 Jan 20;4(1):100336. doi: 10.1016/j.mcpdig.2026.100336 (PMC12937170; doi:10.1016/j.mcpdig.2026.100336)
Supplement: Supplemental Table 2 [file mmc2.docx]

**Supplemental Table2. Characteristics of interventions and controls**

| **Study Year** | **Home INR Device** | **Web-based Platform** | **Remote Monitoring** | **Automated Alerts** | **Mobile Apps** | **Software Driven** | **User Data Entry** | **Embedded Algorithm** | **Physician Involvement** | **Anticoagulation Management** | **Integrated Disease Management** | **Health Education** | **Reinforcing Support** | **Intervention Summary** | **Control Summary** |
| --- | --- | --- | --- | --- | --- | --- | --- | --- | --- | --- | --- | --- | --- | --- | --- |
| 2021^48^ | No – INR measured in clinics or laboratories. | Yes – web-based platform used for data management. | Yes – clinical data reviewed remotely between visits. | Unclear – alert functionality not specified. | Yes – patient-facing smartphone application used. | Yes – software system is the core intervention component. | Yes – patients enter clinical or treatment data. | Partial – structured rules applied but algorithm not fully specified. | Yes – clinicians actively review data and guide management. | Yes – anticoagulation management is a primary intervention focus. | No – anticoagulation-only management. | Yes – structured patient education provided. | Yes – reminders, feedback or ongoing digital follow-up provided. | App-based remote warfarin management with clinician oversight | Usual care with clinic-based INR monitoring |
| 2023^49^ | Unclear – home INR device use not specified. | Yes – web-based platform used for data management. | Yes – clinical data reviewed remotely between visits. | Partial – reminders described but technical details unclear. | Yes – patient-facing smartphone application used. | Yes – software system is the core intervention component. | Yes – patients enter clinical or treatment data. | Partial – structured rules applied but algorithm not fully specified. | Yes – clinicians actively review data and guide management. | Yes – anticoagulation management is a primary intervention focus. | No – anticoagulation-only management. | Yes – structured patient education provided. | Yes – reminders, feedback or ongoing digital follow-up provided. | Social app–supported warfarin management with enhanced monitoring and education | Usual care without social app support |
| 2021^50^ | No – INR measured in clinics or laboratories. | Yes – web-based platform used for data management. | Yes – clinical data reviewed remotely between visits. | Yes – automated reminders or alerts implemented. | Yes – patient-facing smartphone application used. | Yes – software system is the core intervention component. | Yes – clinicians and/or patients enter structured clinical data. | Yes – explicit rule-based or guideline-driven algorithm embedded. | Yes – clinicians actively review data and guide management. | Yes – anticoagulation management is a primary intervention focus. | Yes – integrated AF or multimorbidity management included. | Yes – structured patient education provided. | Yes – reminders, feedback or ongoing digital follow-up provided. | mHealth-supported ABC integrated care pathway for AF | Usual AF care according to local practice |
| 2024^51^ | No – INR measured in clinics or laboratories. | Yes – web-based platform used for data management. | Yes – clinical data reviewed remotely between visits. | Yes – automated reminders or alerts implemented. | Yes – patient-facing smartphone application used. | Yes – software system is the core intervention component. | Yes – patients enter clinical or treatment data. | Partial – structured rules applied but algorithm not fully specified. | Yes – clinicians actively review data and guide management. | Yes – anticoagulation management is a primary intervention focus. | Partial – limited elements beyond anticoagulation. | Yes – structured patient education provided. | Yes – reminders, feedback or ongoing digital follow-up provided. | Alfalfa app providing education, reminders and remote support for AF patients on OAC | Usual care with standard outpatient follow-up |
| 2025^52^ | Unclear – home INR device use not specified. | Yes – web-based platform used for data management. | Yes – clinical data reviewed remotely between visits. | Yes – automated reminders or alerts implemented. | Yes – patient-facing smartphone application used. | Yes – software system is the core intervention component. | Yes – patients and pharmacists enter clinical or treatment data. | Partial – structured rules applied but algorithm not fully specified. | Yes – pharmacist-led with clinician collaboration. | Yes – anticoagulation management is a primary intervention focus. | Partial – limited elements beyond anticoagulation. | Yes – structured patient education provided. | Yes – reminders, feedback or ongoing digital follow-up provided. | Internet + pharmacy anticoagulation care via Alfalfa app with pharmacist support | Usual care with standard hospital follow-up |
| 2009^53^ | Yes – home INR self-testing device used. | Yes – web-based platform used for data management. | Yes – clinical data reviewed remotely between visits. | Partial – reminders described but technical details unclear. | No – intervention delivered without mobile app. | Yes – software system is the core intervention component. | Yes – patients enter clinical or treatment data. | Yes – explicit rule-based or guideline-driven algorithm embedded. | Yes – clinicians actively review data and guide management. | Yes – anticoagulation management is a primary intervention focus. | No – anticoagulation-only management. | Limited – initial or basic education only. | Yes – reminders, feedback or ongoing digital follow-up provided. | Internet-based expert system with supervised INR self-testing and automated dosing | Clinic-based anticoagulation management service |
| 2015^54^ | Yes – home INR self-testing device used. | Yes – web-based platform used for data management. | Yes – clinical data reviewed remotely between visits. | Partial – reminders described but technical details unclear. | No – intervention delivered without mobile app. | Yes – software system is the core intervention component. | Yes – patients enter clinical or treatment data. | Yes – explicit rule-based or guideline-driven algorithm embedded. | Yes – clinicians actively review data and guide management. | Yes – anticoagulation management is a primary intervention focus. | No – anticoagulation-only management. | Limited – initial or basic education only. | Yes – reminders, feedback or ongoing digital follow-up provided. | Telemedicine-guided very low-dose INR self-control in mechanical valve patients | Standard low-dose self-anticoagulation management |
| 2020^55^ | No – INR measured in clinics or laboratories. | Yes – web-based platform used for data management. | No – monitoring limited to in-person visits. | Yes – automated reminders or alerts implemented. | No – intervention delivered without mobile app. | Yes – software system is the core intervention component. | Yes – clinicians enter structured clinical data. | Yes – explicit rule-based or guideline-driven algorithm embedded. | Yes – clinicians actively review data and guide management. | Yes – anticoagulation management is a primary intervention focus. | Yes – integrated AF or multimorbidity management included. | Partial – education prompted but not structured. | Yes – reminders, feedback or ongoing digital follow-up provided. | Computerized CDSS supporting integrated AF and anticoagulation management | Usual AF care without computerized decision support |
| 2019^56^ | Yes – home INR self-testing device used. | Yes – web-based platform used for data management. | Yes – clinical data reviewed remotely between visits. | Partial – reminders described but technical details unclear. | No – intervention delivered without mobile app. | Yes – software system is the core intervention component. | Yes – patients enter clinical or treatment data. | Yes – explicit rule-based or guideline-driven algorithm embedded. | Yes – clinicians actively review data and guide management. | Yes – anticoagulation management is a primary intervention focus. | No – anticoagulation-only management. | Limited – initial or basic education only. | Yes – reminders, feedback or ongoing digital follow-up provided. | Telemedicine-supported home warfarin self-management with predefined criteria | Routine patient self-testing without structured telemedicine support |
| 2024^57^ | Not applicable – NOAC therapy (no INR monitoring). | Yes – web-based platform used for data management. | Yes – adherence data monitored remotely. | Yes – automated reminders or alerts implemented. | Yes – patient-facing smartphone application used. | Yes – software system is the core intervention component. | Yes – patients enter medication or self-measured data. | Partial – structured rules applied but algorithm not fully specified. | Yes – clinicians actively review data and guide management. | Yes – anticoagulation management is a primary intervention focus. | No – anticoagulation-only management. | Yes – structured patient education provided. | Yes – reminders, feedback or ongoing digital follow-up provided. | Smartphone app–based adherence intervention for edoxaban | Usual care without adherence app |
